# Rapid Colorimetric Detection of Wound Infection with a Fluidic Paper Device

**DOI:** 10.3390/ijms23169129

**Published:** 2022-08-15

**Authors:** Javier Hoyo, Arnau Bassegoda, Guillem Ferreres, Dolores Hinojosa-Caballero, Manuel Gutiérrez-Capitán, Antoni Baldi, César Fernández-Sánchez, Tzanko Tzanov

**Affiliations:** 1 Grup de Biotecnologia Molecular i Industrial, Departament d’Enginyeria Química, Universitat Politècnica de Catalunya, Rambla Sant Nebridi 22, 08222 Terrasa, Spain; 2 Unitat de Ferides Complexes, Consorci Sanitari de Terrassa, Hospital de Terrassa, Ctra. Torrebonica, s/n, 08227 Terrassa, Spain; 3 Instituto de Microelectrónica de Barcelona (IMB-CNM), CSIC, Campus UAB, 08193 Bellaterra, Spain; 4 CIBER de Bioingeniería, Biomateriales y Nanomedicina (CIBER-BBN), Jordi Girona 18-26, 08034 Barcelona, Spain

**Keywords:** colorimetric analysis, point-of-care device, myeloperoxidase, chronic wounds, infection biomarker

## Abstract

Current procedures for the assessment of chronic wound infection are time-consuming and require complex instruments and trained personnel. The incidence of chronic wounds worldwide, and the associated economic burden, urge for simple and cheap point-of-care testing (PoCT) devices for fast on-site diagnosis to enable appropriate early treatment. The enzyme myeloperoxidase (MPO), whose activity in infected wounds is about ten times higher than in non-infected wounds, appears to be a suitable biomarker for wound infection diagnosis. Herein, we develop a single-component foldable paper-based device for the detection of MPO in wound fluids. The analyte detection is achieved in two steps: (i) selective immunocapture of MPO, and (ii) reaction of a specific dye with the captured MPO, yielding a purple color with increasing intensity as a function of the MPO activity in infected wounds in the range of 20–85 U/mL. Ex vivo experiments with wound fluids validated the analytic efficiency of the paper-based device, and the results strongly correlate with a spectrophotometric assay.

## 1. Introduction

Wounds that fail to proceed through the normal phases of wound healing in an orderly and timely manner within a period of three months are considered chronic wounds [1]. In developed countries, the demographical rise in the elderly population and the increasing prevalence of lifestyle diseases, such as obesity and diabetes, inevitably increases the burden of chronic wounds. Moreover, the impact of chronic wounds is also adverse in developing countries where foot ulcer problems may drain up to 40% of the available healthcare resources [2,3]. Chronic wound management by healthcare professionals is often challenging, and requires a thorough evaluation of the patient and the wound to guide the subsequent treatment [4]. Bacterial infection is one of the reasons for wound healing failure. Wound infection assessment by the classical clinical signs, such as redness, heat, swelling, and pain revealed to be of limited reliability when contrasted with a time-consuming wound sampling and microbiological testing [5]. Therefore, an early detection of an incipient wound infection by the attending physician is crucial for the choice of an appropriate treatment to avoid wound chronicity [6,7].

Chronic wounds exhibit high levels of senescent cells, proinflammatory cytokines, myeloperoxidase (MPO) and matrix metalloproteinase (MMPs) enzymes, as well as reactive oxidative species [8]. MPO, a heme-containing peroxidase released by neutrophils, catalyzes the oxidation of chloride ions, using hydrogen peroxide as a co-substrate, to produce hypochlorous acid (HClO), a strong bactericide [9]. Since MPO shows about ten times higher activity in infected wounds than in non-infected wounds, it has been postulated as a useful biomarker for wound infection diagnostics [10,11]. Currently, the only commercially available option that physicians can use to quantify MPO is through enzyme-linked immunosorbent assay (ELISA), which is costly and time-intensive. Alternative MPO quantification assays for laboratory testing based on electrochemistry [12,13], fluorescence [14], and enzymatic activity [15] have been reported. However, the economic and social burden of chronic wounds worldwide calls for simple and cheap point-of-care testing (PoCT) devices, allowing health practitioners to achieve fast diagnosis and select appropriate early treatment. Moreover, PoCT devices will facilitate the implementation of affordable screening programs among the population at risk of developing chronic wounds. 

The conventional approach for detecting protein biomarkers by PoCT devices is based on the implementation of sandwich immunoassay formats, where the biomarker present in the tested liquid sample is first captured by an antibody immobilized onto the PoCT substrate, and is afterwards complexed to a detection antibody conjugated to a molecule amenable for detection. In cases where the PoCT device produces a visual signal, the enzyme horseradish peroxidase (HRP), which, under the presence of hydrogen peroxide, oxidizes a chromogenic substrate [16], is the typically used option. However, MPO and HRP can both catalyze similar oxidation reactions using H_2_O_2_, which could cause interferences. Different authors have proposed the development of MPO sensor devices based on alternative methods to the aforementioned sandwich immunoassay. Sensors based on electrochemical detection, where MPO is detected by the reduction of an MPO-oxidized substrate [17,18], or the measurement of hydrogen peroxide consumption [19], have been reported. These sensors are advantageous compared to the immunoassay as they allow the detection of active MPO. Similarly, Ruchira et al. developed an optical sensor to detect MPO by quantifying the degradation of carbon nanotubes induced by the oxidizing activity of this enzyme [20]. However, all these sensor approaches require complex protocols for sample preparation, and specialized electronic devices or supporting reagents, limiting their wider use. 

Paper offers an excellent platform for developing simple, affordable, standalone PoCT devices. Paper is cheap, biodegradable, biocompatible, and is usually white, which is the best platform for colorimetric detection [21]. The application of paper patterning methods allows the generation of channels, reservoirs, and detection zones that confine the flow of fluids within the hydrophilic paper structure. The integration of fluidic technology in PoCT devices enables the sample manipulation and analyte detection in one single component, low reagent usage and short response time, increased sensitivity and specificity, minimum sample requirements, and ease of fabrication [22]. Fluidic paper-based PoCT devices have been developed for the detection of metals [23], drugs [24], microorganisms [25], and bacterial infections [26]. 

In this work, we developed a paper-based device for the rapid detection of MPO in wound fluids (patent pending [27]) that combines the immunocapture of the standard immunoassays but avoids the use of HRP, and the detection step is driven by the MPO enzymatic activity, which catalyzes the oxidative synthesis of a purple dye [11]. To achieve this, we developed a foldable paper device able to separate in space and time the MPO immunocapture and detection steps. The device can be used qualitatively (the user can give a yes or no diagnosis) and quantitatively (the colorimetric readout can be quantified using an office desktop scanner), and the produced color intensity can be correlated to infection-related MPO activities. We developed a foldable device composed of easily manufactured single-patterned paper, and that integrates very economical reagents; the paper component can be used integrated into a reusable transparent plastic cartridge for better handling. The here presented device does not require specific training, the MPO detection can be achieved following simple steps in a similar fashion to lateral flow devices, such as the rapid SARS-CoV-2 antigen test. 

## 2. Results and Discussion

### 2.1. Design of the Paper-Based Fluidic Device 

A paper-based fluidic design was adopted for MPO evaluation via a two-step detection process, namely: (i) immunocapture of MPO from the wound exudate sample by a specific antibody, and (ii) colorimetric detection based on enzymatic oxidation of chromogenic substrates by the immunocaptured MPO. Therefore, the device should consider these two steps to establish a pattern that avoids the flow in an undesired direction. Fluidic devices that prevent flow in an undesired direction rely on valves, different channel lengths, or the addition of revealing solutions [28,29,30]. Alternatively, we engineered a fluidic paper design, simple for manufacturing and use, that allows the separation in space and time of two sequential events for the detection of MPO. This separation allows for the implementation of complementary biomarker detection reactions to increase the detection specificity while avoiding possible interferences. 

**Figure 1:**
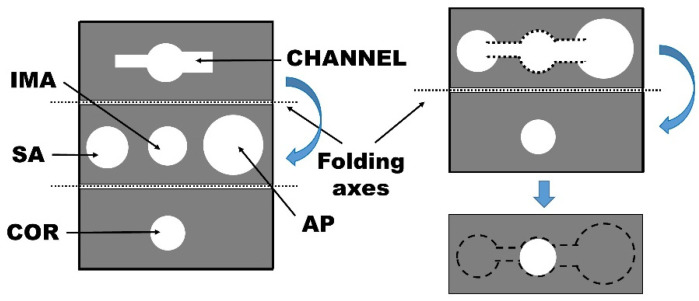
Scheme of the fluidic device including the folding axes (**left**) and folding procedure (**right**). The areas defined are: SA, sample addition area; IMA, immobilization of anti-MPO antibody area; AP, absorbent pad area; and COR, immobilization of the chromogenic substrate area.

The fluidic pattern defines the following areas: (i) a sample application area (SA), (ii) channel, (iii) immobilized antibody zone (IMA), (iv) an area for immobilization of the chromogenic substrates (COR), and (v) an absorbent pad area (AP). SA, IMA, and AP are placed in the same paper plane, whereas the channel and COR are located in an independent foldable part of the device without direct connection with the aforementioned areas (Figure 1). 

**Figure 2:**
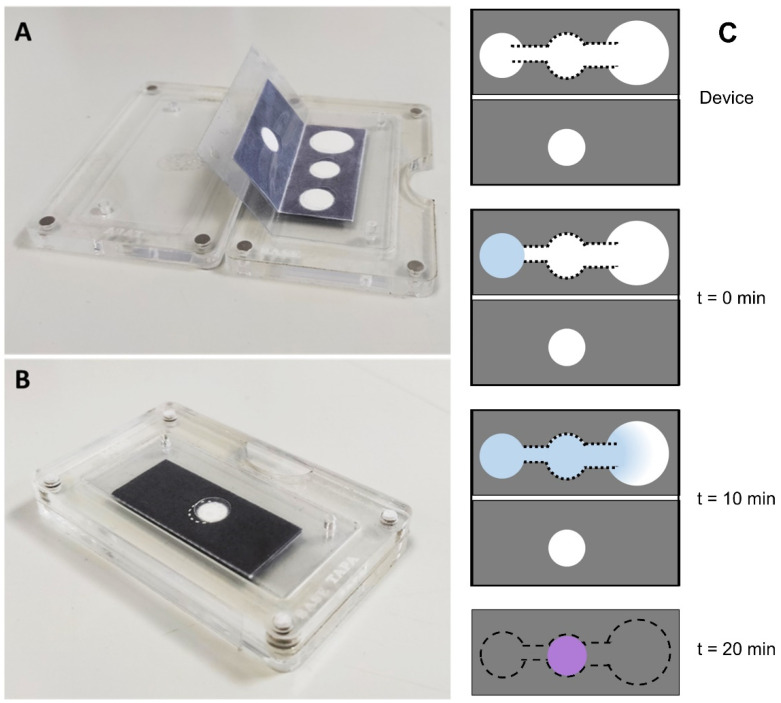
Reusable plastic cartridge to encapsulate the paper-based fluidic device in (**A**) open position and (**B**) closed position. (**C**) Scheme of the analyte fluid movement on the paper device.

The fluidic device is a single-cellulose sheet that is folded as follows: (i) Before sample addition, connect SA, IMA, and AP in the x–y plane through the channel and allow the flow of the sample to the IMA zone, where MPO is retained due to the specificity of the antibody. (ii) After sample application, and once the flow reaches the end of the AP, the reaction in the IMA zone is considered completed; connecting the IMA and COR zones in the z direction puts into contact the chromogenic reagents immobilized in the COR area with the immobilized MPO in the IMA zone, triggering the colorimetric reaction. The SA, IMA, and AP zones are placed separately to avoid unspecific and undesired interactions between components of the liquid sample and the reagents immobilized on the cellulose support during the device fabrication. The channels are designed to allow a correct sample flow and achieve the immunocapture in an optimized reaction time. The IMA and COR zones are placed in separate zones to avoid the reaction of the COR reagents with the antibodies, or with biomarkers different from MPO that are in the sample. Such biomarkers, although not being of interest for detection, may contribute to the colorimetric reaction, thus inducing false results. Once the immunocapture is achieved, the device is folded to put into contact IMA and COR, which have to remain in contact for 10 min to fully react and reveal the purple color indicative of MPO presence. To systematically evaluate the performance of the fluidic device, we fabricated a homemade reusable plastic cartridge in which the paper piece is encapsulated (Figure 2A) and then the cartridge is closed (Figure 2B) to trigger the colorimetric reaction.

The herein developed fluidic device could also be used for qualitative analysis of other biomarkers, following the same rationale of a selective immunocapture in the IMA zone and an appropriate revealing solution in the COR zone. The fluidic device will be further tested for qualitative analysis of bacterial infection in chronic wounds through the analysis of the MPO biomarker in wound fluids. 

### 2.2. Fluidic Device In Vitro Validation

#### 2.2.1. Validation of the Fluidic Device with Commercial MPO

The activity of commercial pure MPO (120 U/mL) was evaluated following a method, as described in [8]. Thereafter, different dilutions corresponding to the MPO activities found in infected wound fluids [10,11] were prepared to validate the fluidic device.

**Figure 3:**
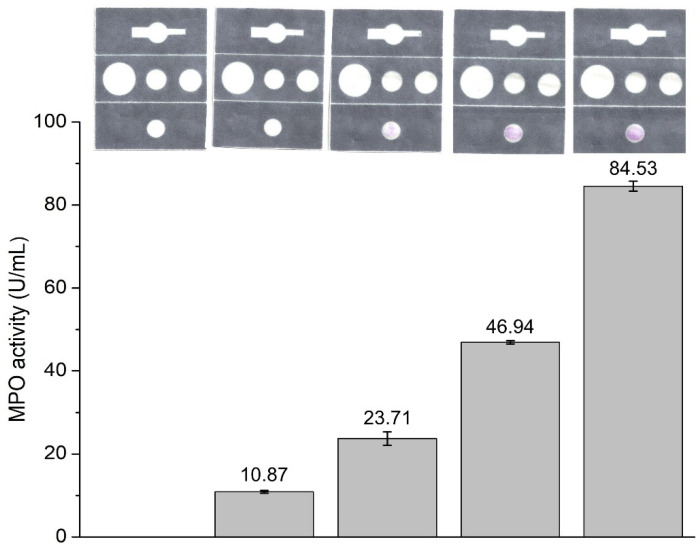
Fluidic paper device tested at different MPO activity (N = 5). Each sample was analyzed using the fluidic device (images) and spectrophotometrically (numerical values in U/mL of MPO in solution).

The functionalized fluidic device was folded to connect the SA, IMA, and AP zones, then the sample solution (10 µL) and PBS (50 µL) were added, in that order, to the SA zone, initiating the liquid flow through the connected IMA and AP areas. After 10 min to ensure reaction between the immobilized antibody and MPO, the fluidic device was folded again to establish contact between the IMA and COR zones until a stable purple color is obtained in the COR area (~10 min). The color intensity is a function of the activity of MPO in the tested sample (Figure 3).

#### 2.2.2. Semi-Quantitative Analysis of Color Intensity

**Figure 4:**
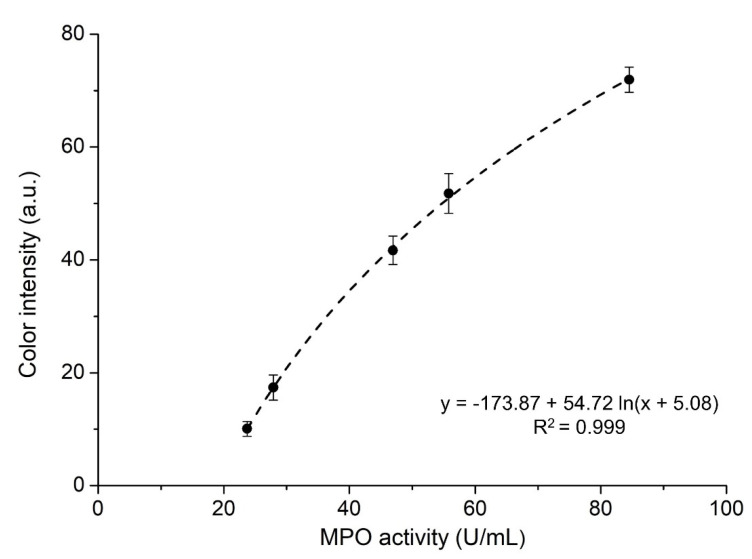
Color intensity of COR zone vs. MPO activity in the standard solutions quantified using spectrophotometry (N = 5).

The developed PoCT is intended to be used by unskilled personnel, on-site, and without the aid of any other instrument. A proportional correlation between the color intensity and the presence of bacterial infection is highly relevant for health practitioners to select an appropriate treatment. The average color intensity of the COR zone was evaluated by converting it to a grey scale (0–255) and considering the intensity of all the pixels comprised in that zone (see Section 3.4). The results (Figure 4) indicate a logarithmic relationship between the color intensity and the presence of MPO in the sample (5 to 85 U/mL) at a concentration range typical for infected wound fluids [10]. At higher MPO concentrations, the immobilized antibody is unable to capture all MPO, which, in addition to saturating the color in the COR zone, would invalidate the results. The fluidic device could be further tuned for detecting larger MPO quantities in other body fluids by adjusting the quantity of the immobilized reagents and the size of the reaction zones. 

#### 2.2.3. Evaluation of Cross-Reactions 

Hemoglobin (Hb) present in wounds could interfere with MPO detection due to its peroxidase activity [31]; thus, possible cross-reactions leading to false positives were evaluated. Several testing solutions were prepared as described in Section 3.2 using Hb instead of MPO. A range (1–70 µM) of Hb concentrations described for infected and non-infected wound fluids [31] was evaluated. The absence of color change (Figure S1) indicated that even at concentrations (70 µM) ten times higher than those reported [31] for wound fluids, Hb did not produce any cross-reactions with the fluidic device compounds. The selective step in the IMA zone prevented the occurrence of undesired side-reactions.

### 2.3. Ex Vivo Validation of the Foldable Fluidic Device with Wound Fluids

**Figure 5:**
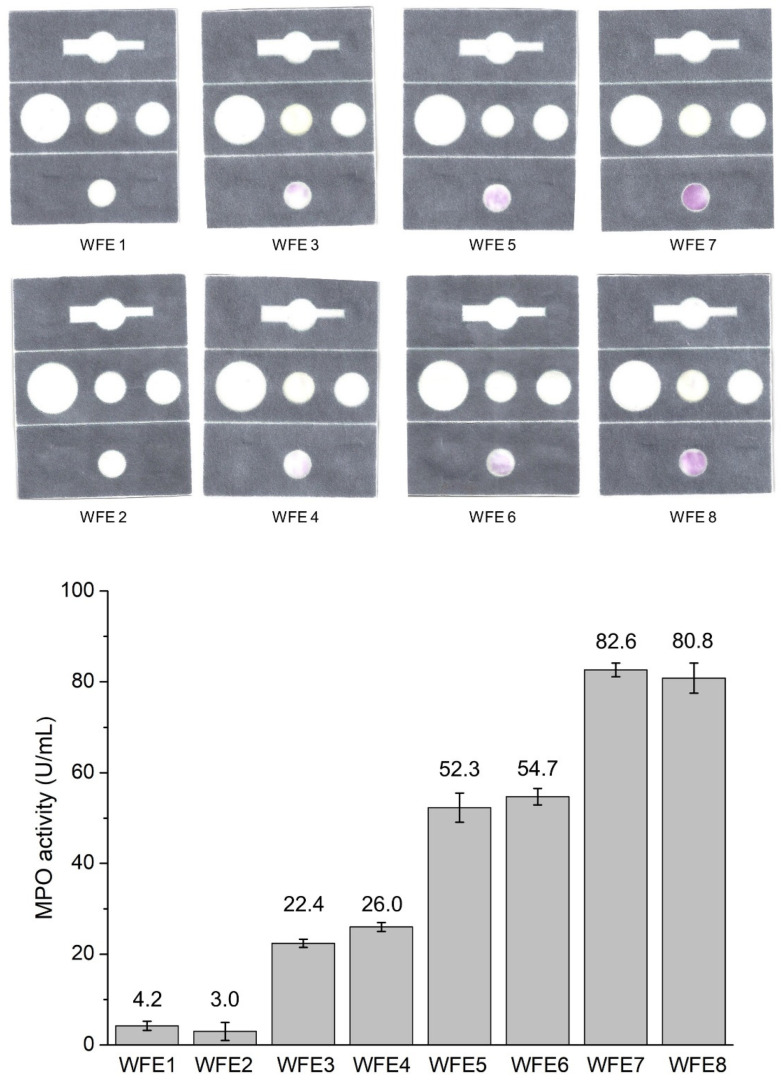
Ex vivo validation of the fluidic paper device with wound fluid extracts (WFE) at different infection levels that correlate with the MPO activity (N = 3). Each WFE was analyzed using the fluidic device (images) and spectrophotometrically (numerical values in U/mL of MPO in the WFE).

The fluidic device was tested with samples of real wound fluid extracts (WFE). Anonymized samples from different patients at different wound healing stages were collected and analyzed using both the spectrophotometric standard method for MPO quantification in wound fluids and using the fluidic paper device. Two representative fluidic devices from each level of infection of different samples are presented in Figure 5. Both techniques detected similar MPO contents for each sample regardless the level of infection of the WFE. These experiments are intended to evaluate the device in the presence of all the components of natural wound fluids. The results indicate that the other components have no effect on the colorimetric assay, and that MPO alone is responsible for the color change. Additionally, the comparison with the spectrophotometric analysis confirms that both analytical techniques render similar results: the fluidic device is faster and without need of external instruments. Therefore, the results confirm that the fluidic paper device for MPO detection is suitable for fast on-site qualitative evaluation at the level of wound infection. 

## 3. Materials and Methods

### 3.1. Materials

Polyethyleneimine (PEI), branched, 50%, 750,000 Da; m-phenylenediamine (mPD); p-phenylenediamine (pPD); glucose; 2,2′-azino-bis(3-ethylbenzothiazoline-6-sulfonic acid) diammonium salt (ABTS); dimethyl sulfoxide (DMSO); bovine serum albumin (BSA); Tween^®^ 20 and Whatman™ chromatography 1CHR paper; hemoglobin (Hb); glutaraldehyde, 25% (*w*/*w*) aqueous solution; and guaiacol and glucose oxidase (GOX) from *Aspergillus niger* were purchased from Sigma-Aldrich^®^ (Madrid, Spain). Myeloperoxidase (MPO) from human leukocytes was purchased from Planta Natural Products (Vienna, Austria). PERDROGENTM 30% H_2_O_2_ (*w*/*w*) was obtained from Fluka (Madrid, Spain), phosphate buffered saline tablets were purchased from Fisher Bioreagents (Madrid, Spain), and rabbit polyclonal anti-myeloperoxidase antibody was obtained from Abcam (Cambridge, UK). 

### 3.2. Fluidic Paper Device Fabrication and Functionalization

Hydrophobic wax was printed on chromatographic Whatman™ (Sigma-Aldrich^®^, Madrid, Spain) chromatography 1CHR paper using a Xerox 8570 (Madrid, Spain) printer to establish the optimized fluidic pattern. Then, the paper was placed, for 2 min, in the laboratory oven at 150 °C to allow the penetration of the hydrophobic wax through the whole paper thickness, thus achieving efficient 3-dimension (3D) liquid flow control via the hydrophobic pattern. First, the MPO antibody was covalently immobilized on the immobilization of anti-MPO antibody (IMA) area of the paper device (Figure 1). Solution of low-molecular-weight chitosan (1.5 µL, 0.25 mg/mL) in 0.1 M acetic acid was added and left to dry at room temperature (RT, 22 °C) for 10 min. Afterwards 1.5 µL of 2.5% (*v*/*v*) glutaraldehyde in PBS was added to the IMA area and the device was incubated for 2 h at RT under humid conditions. After the incubation, the IMA area was washed with 2 mL of deionized (DI) water and left to dry at RT for 20 min. Anti-MPO solution (1 µL, 1 mg/mL) was added to the glutaraldehyde-activated IMA area, and the device was incubated again for 2 h at RT under humid conditions. Afterwards, the IMA area was washed with 2 mL of DI water and left to dry at RT for 20 min. Following antibody functionalization, all the areas, except for the absorbent pad (AP) and that for the immobilization of the chromogenic substrates (COR), were soaked with 200 mM phosphate buffer solution at pH 6.5 containing 0.5% (*w*/*v*) BSA, 0.1% (*w*/*v*) Tween^®^, and 5 mM glucose. The device was then incubated for 1 h at room temperature under humid conditions, and dried thereafter at RT for 1 h. Finally, the COR area was functionalized by first adding 1.5 µL of GOX solution (2 mg/mL), and drying at RT for 10 min. Once dried, 1.5 µL of the substrate solution for colorimetric detection (see Section 3.4) was added and left to dry at RT for 10 min. The dried functionalized paper devices were stored at 4 °C until use. 

### 3.3. MPO Activity Evaluation

The oxidation activity of pure enzyme or wound exudates was measured with the guaiacol assay [32]. Enzyme standards containing 5 U/mL up to 85 U/mL MPO were prepared for calibration, and the wound exudate extracts were diluted eight times in PBS. The reaction was carried out in PBS solution (0.1 M, pH 7.4) in the presence of guaiacol (10 mM), hydrogen peroxide (0.2 mM), and 4% (*v*/*v*) of the enzyme/extract solution. The increase in absorbance at 470 nm was measured for 10 min using a microplate reader (Infinite M200, Tecan, Salzburg, Austria). 

### 3.4. MPO Colorimetric Evaluation with the Fluidic Device

#### 3.4.1. MPO Colorimetric Detection

The colorimetric detection of MPO by the developed fluidic paper device was based on MPO-catalyzed oxidative synthesis of a dye [11]. In brief, a solution containing ABTS (2 mM), m-phenylenendiamide (2 mM), and PEI (10% (*w*/*v*)) prepared in PBS was used. The oxidation of the substrates ABTS and m-phenylenendiamide by MPO in the presence of hydrogen peroxide yields a purple dye clearly distinguishable with the naked eye. Hydrogen peroxide, the co-substrate of MPO, was generated in the system by the conversion of glucose by glucose oxidase (GOX) from *Aspergillus niger*. As mentioned above, 1.5 µL GOX solution (2 mg/mL) was applied onto the COR area and, once dried, 1.5 µL substrate solution was added and dried. The fluidic device was operated as described in Section 2.1, and the color appearing in the COR zone was analyzed using a digital photography scanner (HP Photosmart 509, Hewlett-Packard, Madrid, Spain). 

In order to systematically analyze the shade of the purple color, the devices were scanned and transformed from color images to grey images to obtain a grey scale from 0 (black) to 255 (white). The method counts the grey intensity of each pixel of the COR zone and from this deduces the average grey intensity of the COR zone. ImageJ 1.7.0 was used to draw a circle (same dimensions for all the samples) that was positioned on the COR zone in such a way that is inscribed within the COR zone. ImageJ was then used to render a histogram of the different grey shades present in the circle, and displays the average COR shade. Therefore, the average grey shade (value from ImageJ) of each sample was subtracted from the average of the grey shade of the samples, with no presence of MPO (247). The value 247 was used to standardize the results due to the fact that chromatographic paper is not pure white (255). 

#### 3.4.2. Sample Preparation for Validation of the Fluidic Device

Pure MPO, or Hb in cases of possible cross-reaction evaluation, was diluted in PBS to different final activities. First, 10 µL enzyme dilution was applied onto the SA zone of the fluidic device at room temperature, followed by 50 µL PBS. For the evaluation of ex vivo wound fluids, the wound exudates were extracted from waste dressings used on chronic wounds from different etiologies, such as venous leg ulcers, diabetic ulcers, and pressure wounds, kindly provided by Hospital de Terrassa (Spain). The dressings were soaked in PBS (5 mL buffer per gram of dressing) for 10 min, then the mixture was vortexed for 3 min, and centrifuged for 5 min at 4000 rpm. Subsequently, the supernatants were collected and centrifuged one more time for 5 min at 4000 rpm to remove the remaining fibers and sludge from the liquid. Finally, 10 µL wound exudate was loaded onto the SA area of the fluidic device, followed by 50 µL PBS to start the flow through the device. 

## 4. Conclusions

In summary, we have developed a foldable paper-based fluidic device for the detection of MPO in wounds that meets the requirements of a PoCT device to detect wound infection and prevent the appearance of chronic wounds. The device produces a clear, purple, visible readout when it detects infection-related MPO activity. If necessary, the color intensity can be simply quantified, and is correlated with MPO activities within the range of 20–85 U/mL; thus, the device use can be at both qualitative and quantitative levels. The MPO detection is achieved following simple steps, namely, the addition of a liquid sample and buffer, and the folding of the device. We have achieved a simple and cheap device that can be easily mass-produced. Therefore, this paper-based fluidic device would allow the implementation of affordable screening programs among the population endangered by wound infections, and for wound testing in places where more sophisticated analytical techniques are of limited availability.

## 5. Patents

The authors have filed a patent pending entitled “A foldable fluidic device and method for biomarker detection in body fluids” to PCT/EP2021/059153.

## Data Availability

Not applicable.

## Supplementary Materials

The supporting information can be downloaded at: https://www.mdpi.com/article/10.3390/ijms23169129/s1.
