# Supplementary material for: Rapid Colorimetric Detection of Wound Infection with a Fluidic Paper Device
Source: Int J Mol Sci. 2022 Aug 15;23(16):9129. doi: 10.3390/ijms23169129 (PMC9408953; doi:10.3390/ijms23169129)
Supplement: Supplementary file 1 [file ijms-23-09129-s001.zip › ijms-1860148-supplementary.pdf]

International Journal of *Molecular Sciences*

MDPI

Communication

Rapid Colorimetric Detection of Wound Infection with a Fluidic Paper Device

Javier Hoyo [1], Arnau Bassegoda [1], Guillem Ferreres [1], Dolores Hinojosa-Caballero [2], Manuel Gutiérrez-Capitán [3], Antoni Baldi [3], César Fernández-Sánchez [3,4] and Tzanko Tzanov [1,*]

[1] Grup de Biotecnologia Molecular i Industrial, Departament d'Enginyeria Química, Universitat Politècnica de Catalunya, Rambla Sant Nebridi 22, 08222 Terrasa, Spain
[2] Unitat de Ferides Complexes, Consorci Sanitari de Terrassa, Hospital de Terrassa, Ctra. Torrebonica, s/n, 08227 Terrassa, Spain
[3] Instituto de Microelectrónica de Barcelona (IMB-CNM), CSIC, Campus UAB, 08193 Bellaterra, Spain
[4] CIBER de Bioingeniería, Biomateriales y Nanomedicina (CIBER-BBN), Jordi Girona 18-26, 08034 Barcelona, Spain
* Correspondence: tzanko.tzanov@upc.edu

Citation: Hoyo, J.; Bassegoda, A.; Ferreres, G.; Hinojosa-Caballero, D.; Gutiérrez-Capitán, M.; Baldi, A.; Fernández-Sánchez, C.; Tzanov, T. Rapid Colorimetric Detection of Wound Infection with a Fluidic Paper Device. *Int. J. Mol. Sci.* **2022**, *23*, 9129. https://doi.org/10.3390/ijms23169129

Academic Editors: Giorgio Rispoli and Pierpaolo Greco

Received: 26 July 2022
Accepted: 11 August 2022
Published: 15 August 2022

Publisher's Note: MDPI stays neutral with regard to jurisdictional claims in published maps and institutional affiliations.

Copyright: © 2022 by the authors. Submitted for possible open access publication under the terms and conditions of the Creative Commons Attribution (CC BY) license (https://creativecommons.org/licenses/by/4.0/).

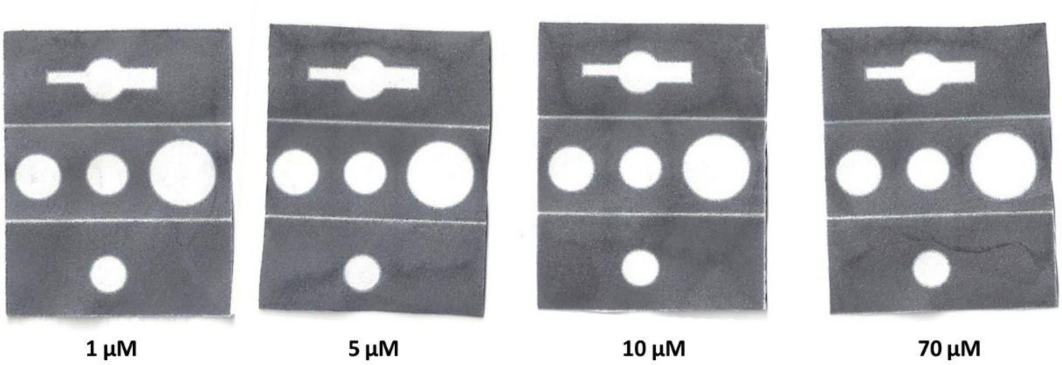

Figure S1. Fluidic paper device tested at several concentrations of Hb for cross-reactions with the fluidic device (N=3).
